# New Strategies for Volume Control in Patients with Diabetes Mellitus, a Narrative Review

**DOI:** 10.3390/pharmaceutics14081569

**Published:** 2022-07-28

**Authors:** Alexandre O. Gérard, Audrey Laurain, Antoine Sicard, Diane Merino, Atul Pathak, Milou-Daniel Drici, Guillaume Favre, Vincent L. M. Esnault

**Affiliations:** 1 Nephrology-Dialysis-Transplantation Department, Pasteur Hospital, Université Côte d’Azur, 06001 Nice, France; gerard.a@chu-nice.fr (A.O.G.); laurain.a@chu-nice.fr (A.L.); sicard.a@chu-nice.fr (A.S.); favre.g@chu-nice.fr (G.F.); 2 Pharmacology Department, Pasteur Hospital, Université Côte d’Azur, 06001 Nice, France; merino.d@chu-nice.fr (D.M.); pharmacovigilance@chu-nice.fr (M.-D.D.); 3 Centre Hospitalier Princesse Grace, 98000 Monaco, Monaco; atul.pathak@chpg.mc

**Keywords:** diuretics, aldosterone breakthrough, heart failure, congestion, kidney, proteinuria, physiology

## Abstract

Sodium is reabsorbed all along the renal tubules. The positive impacts of sodium-glucose cotransporter-2 inhibitors (SGLT2i), angiotensin receptor neprilysin inhibitor (ARNI) and mineralocorticoid receptor antagonists (MRA) on hard renal and/or cardiac endpoints calls for the role of diuretics in nephroprotection and cardioprotection in patients with diabetes mellitus to be reviewed. Here, we review: (a) the mechanisms of action of the available natriuretics; (b) the physiological adaptations to chronic loop diuretic usage that lead to increased sodium reabsorption in the proximal and distal convoluted tubules; (c) the physiology of sodium retention in patients with diabetes mellitus; and (d) the mechanisms of aldosterone breakthrough. We show the rationale for combined diuretics to target not only the loop of Henle, but also the proximal and distal convoluted tubules. Indeed, higher residual proteinuria in patients treated with renin-angiotensin-aldosterone system (RAAS) blockers portends poorer renal and cardiovascular outcomes. Diuretics are known to optimize the reduction of proteinuria, in addition to RAAS blockers, but may favor aldosterone breakthrough in the absence of MRA. The aim of our study is to support a combined diuretics strategy to improve the management of patients with diabetes mellitus and chronic kidney disease or heart failure.

## 1. Introduction

Diabetes mellitus (DM) is often complicated by diabetic kidney disease (DKD), which is the most frequent etiology of end-stage renal disease [1]. DM also contributes to a worldwide rapid increase of cardiovascular morbimortality [2]. The vicious circles between type 2 diabetes (T2D), chronic kidney disease (CKD) and heart failure (HF) are underpinned by several common pathophysiological mechanisms, including sodium retention and the upregulation of the renin-angiotensin-aldosterone system (RAAS) [3]. This suggests that both control of volume overload and optimal RAAS blockade might be pivotal for the management of patients with T2D. Indeed, many drugs that showed a significant benefit in renal and/or cardiovascular outcome trials in addition to conventional RAAS blockers (i.e., angiotensin converting enzyme inhibitors -ACEI- or angiotensin receptor blockers -ARB-) have a natriuretic effect, including sodium–glucose cotransporter-2 inhibitors (SGLT2i) [4], angiotensin receptor-neprilysin inhibitor (ARNI) [5] and mineralocorticoid receptor antagonists (MRA) [6], although most patients involved in these trials had already received diuretics. In fact, higher residual proteinuria in patients treated with RAAS blockers has been reported to lead to poorer renal and cardiovascular outcomes. Nonetheless, as emphasized by a recent review [7], diuretics are known to optimize the reduction of proteinuria, in addition to RAAS blockers. This effect, added to the benefit of salt restriction, is stronger than double RAAS blockade [8] and is required to slow the decrease of glomerular filtration rate (GFR).

Even more than tight glycemic control per se, the choice of glucose-lowering drugs may influence clinical outcomes [9]. Indeed, the management of T2D has been revolutionized by SGLT2i [10]. Their benefits extend well beyond their mere glucose-lowering effect, only marginal in patients with DM and CKD. Indeed, SGLT2i are associated with improved cardiovascular and renal outcomes, even in patients with HF and/or CKD without diabetes [11,12]. The magnitude and speed of the decrease in cardiovascular and renal events in patients treated with SGLT2i highlights the importance of volume control [13].

ARNI decrease both hospitalization for HF and cardiovascular mortality, compared to angiotensin receptor blockade alone, irrespective of DM status. This effect was observed in patients featuring HF with a reduced [5] and maybe also preserved [14] ejection fraction. Although most patients in these studies had already received other diuretics, increased levels of natriuretic peptides helped to prevent congestive HF.

Salt restriction and diuretics may foster aldosterone breakthrough in patients treated with ACEI or ARB [15]. This could contribute to the residual renal and cardiovascular risk in patients with DM treated with conventional RAAS blockers. Therefore, MRA may be required for optimal RAAS blockade. Indeed, spironolactone and eplerenone added to conventional RAAS blockade reduce the morbi-mortality of patients with heart failure with a reduced ejection fraction [16,17,18]. Finerenone, a non-steroidal mineralocorticoid receptor antagonist (MRA), also added to RAAS blockade, reduces major renal and cardiac events in patients with DKD [19,20].

**Figure 1:**
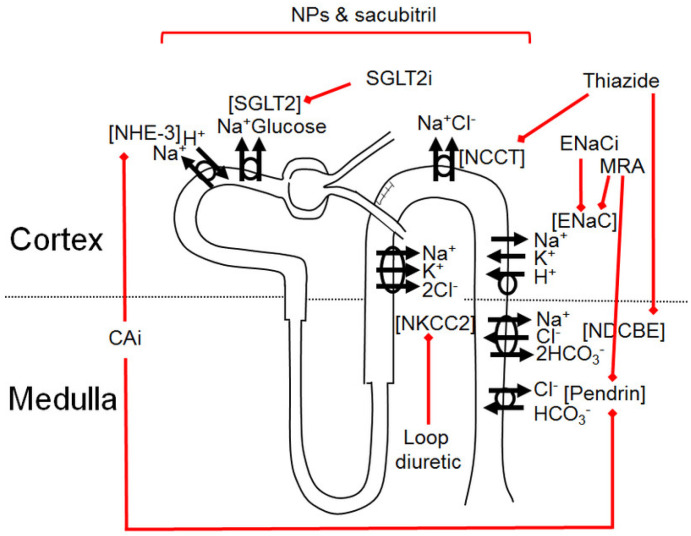
Diuretics and their action in the nephron. Almost all components of the tubule involved in sodium retention can be targeted by existing diuretics, paving the way to sequential nephron blockade. The proximal tubule is targeted by SGLT2 inhibitors, natriuretic peptides (increased in patients treated with sacubitril), and carbonic anhydrase inhibitors. The loop of Henle and the proximal and distal convoluted tubule are targeted by loop and thiazide diuretics, respectively. Sodium retention in the collecting duct is mainly hindered by mineralocorticoid receptor antagonists and ENaC inhibitors. [SGLT2]: sodium–glucose cotransporter-2, [NHE-3]: Na^+^/H^+^ exchanger type 3, [NKCC2]: sodium–potassium–chloride cotransporter type 2, [NCCT]: sodium–chloride cotransporter, [NDCBE]: Na^+^-driven Cl^−^/HCO_3_^−^ exchanger, [ENaC]: epithelial sodium channel, SGLT2i: sodium–glucose cotransporter-2 inhibitors, NPs: Natriuretic Peptides, MRA: Mineralocorticoid Receptor Antagonists, ENaCi: Epithelial sodium channel inhibitor, CAI: carbonic anhydrase inhibitor.

In our study, we reviewed the rationale for combining different classes of diuretics (Figure 1, Table 1) for renal and cardiovascular protection in patients with DM, since the blockade of a unique renal sodium transporter leads to increased sodium reabsorption in other parts of the tubules.

## 2. Rationale for Diuretics in Diabetes Mellitus

### 2.1. Sodium Retention in Diabetes Mellitus

Sodium retention is a key player in numerous pathological conditions, most of which are frequently diagnosed in patients with DM. Increased sodium retention and hypervolemia strongly contribute to hypertension in patients with DM [21]. Increased glomerular filtration of glucose upregulates glucose and sodium cotransport in the proximal tubule, through SGLT channels, leading to chronic mild volume expansion. In addition, sodium retention is further increased in patients with DM treated with insulin, highlighting the intimate bond between sodium and glucose handling [22]. Hyperinsulinemia, secondary to insulinoresistance in T2D, has an antinatriuretic effect mediated by the sympathetic nervous system [23] and RAAS activation [24], which promotes salt retention through hemodynamic changes and increased reabsorption [25]. Vasopressin may also contribute to volume expansion [26].

More recently, magnetic resonance imaging analysis confirmed that patients with T2D exhibit increased skin and muscle sodium content, which is related to organ damage [27]. In addition to hypertension and HF, fluid overload directly increases intraglomerular pressure, worsening proteinuria in DKD.

### 2.2. Limitations of Monotherapies

In physiology and pharmacology, escape to a monotherapy acting on a single pathological pathway is an expected outcome. The doxazosin arm of ALLHAT study was terminated early because of a 25% higher rate of combined cardiovascular events, including congestive HF. Indeed, alpha-blockers monotherapy induce sodium retention and adrenergic and renin-angiotensin-aldosterone system activations, contributing to increased risk of congestive HF. ALLHAT study also showed that chlorthalidone was superior to ACEI for the prevention of major cardiovascular disease events, [28] suggesting that volume control is required for cardiovascular protection in this high-risk population whose high salt intake could have blunted most of ACEI’s beneficial effects [29].

RAAS blocking agents were mainly used in association with diuretics in cardiovascular outcome trials. PROGRESS trial showed that only patients receiving both ACEI and diuretic were protected against recurrent stroke [30].

## 3. Available Natriuretic Drugs and Synergies

### 3.1. Carbonic Anhydrase Inhibitors

Carbonic anhydrase inhibitors (CAI) indirectly inhibit Na/H exchanger type 3 (NHE-3) at the apical membrane of proximal convoluted tubule and have a modest effect on distal tubule, where they inhibit pendrin (a HCO3/Cl exchanger) [31]. The overall impact on blood pressure is disappointing because bicarbonate loss promotes carbonic anhydrase activation, leading to early breakthrough, enhanced by increased downstream sodium reabsorption. However, as pendrin is one of thiazide resistance mechanisms, [32] a sequential nephron blockade with CAI associated with thiazide diuretics and ENaC inhibitors may lead to stronger sodium depletion compared to monotherapies. However, other treatments with excellent long-term tolerance profiles are available to chronically decrease sodium reabsorption in proximal convoluted tubule. Indeed, CAI-induced acidosis is incompatible with long-term treatments.

### 3.2. Sodium-Glucose Cotransporter-2 Inhibitors (SGLT2i)

SGLT2i act by inhibiting Na-Glucose cotransport in proximal convoluted tubule. After starting SGLT2i, a clinically significant natriuresis is exhibited with rapid body weight loss, mimicking thiazide-induced volume depletion, and with dose-dependent decrease in blood pressure [33]. When GFR is below 60 mL/min/1.73 m^2^, SGLT2i have a marginal effect on glycemia, but still exert clinically significant antihypertensive activity: this modest inhibition of sodium reabsorption in proximal convoluted tubule is still significant in these patients. While GFR decreases, more glucose is filtered in every single remaining nephron and osmotic-driven sodium secretion is increased in the tubule [34]. SGLT2i have different selectivity for renal SGLT2 over gut SGLT1, with canagliflozin having the lowest selectivity index (250-fold) [35]. The contribution of extra-renal sodium loss to canaglifozin-induced blood pressure decrease remains unknown. Compared to other diuretics, SGLT2i have very distinctive features, as they do not stimulate sympathetic activity, and confer a lower risk of ionic disorders, while also lowering serum uric acid [36].

**Figure 2:**
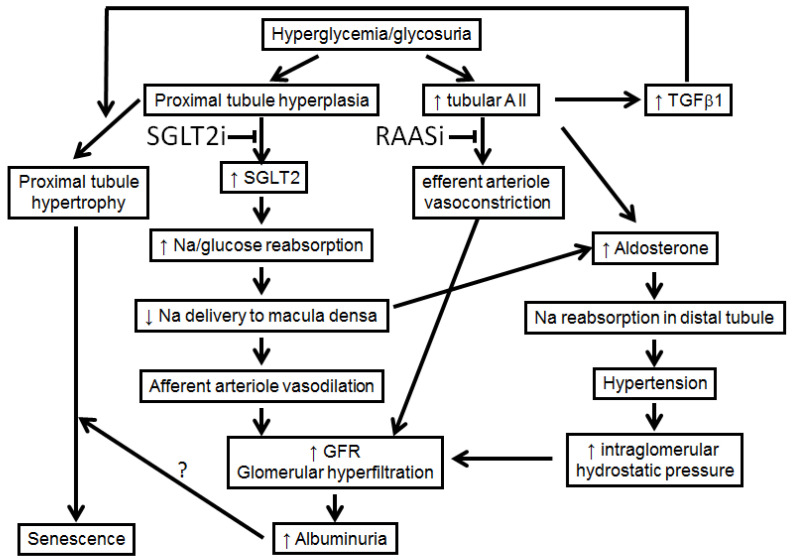
SGLT2 inhibitors and RAAS blockers are required to control glomerular hyperfiltration and albuminuria in diabetes. SGLT2 inhibitors reverse afferent arteriole vasodilation thanks to their proximal natriuretic effect, while RAAS blockers induce efferent arteriole vasodilation. The resulting drop in intraglomerular pressure decreases the GFR in the short term, but is paramount to long-term nephroprotection. AII: angiotensin II, SGLT2: sodium–glucose cotransporter-2 SGLT2i: sodium–glucose cotransporter-2 inhibitors, RAASi: renin–angiotensin–aldosterone system inhibitors, GFR: glomerular filtration rate; TGFβ1: transforming growth factor beta 1.

At an early stage of diabetes (mainly type 1), proximal convoluted tubule hyperplasia with increased expression of SGLT2 contributes to increased sodium reabsorption. Although assessing the precise diuretic effect of SGLT2i is methodologically challenging, [36] SGLT2i decrease sodium reabsorption in proximal convoluted tubule and increase sodium delivery to macula densa, leading to the re-activation of tubuloglomerular feedback and afferent arteriole vasoconstriction, thus decreasing intraglomerular pressure [34] (Figure 2). However, this mechanism is unlikely to fully explain the decreased intraglomerular pressure in patients with T2D and advanced CKD, due to their impaired neurohormonal response to tubuloglomerular feedback. In these patients, SGLT2i may rather exert a synergistic effect with RAAS blockers on efferent glomerular arterioles, most likely by controlling volume overload, just like they prevent congestion in heart failure [37]. As fluid is left in the tubular lumen, SGLT2i might also decrease intraglomerular pressure by increasing the hydrostatic back pressure in the Bowman’s space [38]. Moreover, increased sodium delivery to distal tubule has a kaliuretic effect, improving tolerance of RAAS inhibitors [34].

The abolition of glucose-reabsorption-induced hyperfiltration causes an initial transient decrease in GFR, subsequently offset by increased sodium reabsorption along the loop of Henle [34]. This suggests a potential synergistic effect of SGLT2i and LD on volume overload. Conversely, SGLT2i limit proximal tubule hyperactivity resulting from increased sodium reabsorption, one of the LD resistance mechanisms.

In DAPA-HF, dapagliflozin decreased the risk of worsening HF or cardiovascular death, irrespective of DM status [39], independently of MRA or ARNI (McMurray JJ, ESC 2019, Paris, France), suggesting that sequential sodium reabsorption blockade is required to prevent escape from natriuretic monotherapies.

However, beyond decreased intra-glomerular pressure, the benefit of SGLT2 inhibitors is most likely explained by pleiotropic mechanisms, including blood pressure reduction as well as favorable effects on vascular function, reduction in tubular workload and hypoxia and metabolic effects [40].

### 3.3. Loop Diuretics

LD are secreted into tubular lumen through the organic anion secretory system of the proximal convoluted tubule. They inhibit the NKCC2 symporter of the ascending limb of the loop of Henle, where up to 20–25% of the filtered sodium is reabsorbed. NKCC1 receptor’s inhibition may contribute to the vasodilatory effect on vascular muscles. Enhanced secretion of renal prostaglandins further contributes to vasodilation, especially on afferent arteriole, resulting in increased glomerular sodium filtration.

In spite of powerful acute natriuretic activity, numerous mechanisms can contribute to treatment escape to LD, irrespective of the administration regimen.

(i) The short half-life of LD underlies the acute braking phenomenon, [41] with a decline in the filtered amount of sodium and an increase in proximal sodium reabsorption through glomerulotubular balance.
(ii) After several days, sodium depletion and rising prostacyclins activate RAAS. LD inhibit NKCC2 on macula densa, resulting in the inhibition of the tubuloglomerular feedback, [42,43] preventing afferent arteriole vasoconstriction. This may impair nephroprotection in patients with T2D when LD are used alone. RAAS inhibitors can prevent these important mechanisms of LD resistance and deleterious neuroendocrine activation [44]. Conversely, LD are synergistic with all drugs blocking RAAS, enhancing their antiproteinuric effect through sodium depletion [8,45].
(iii) The long-term effect of LD is further chronically limited by the compensatory hypertrophy of early distal convoluted tubule. Thus, LD synergy with thiazides is expected. Thiazides limit the enhanced distal reabsorption of sodium and the prolong LD effect with their longer half-life, as a perfect example of sequential nephron blockade. However, the association of LD and thiazides exposes patients to a risk of hypokaiemia, which can be prevented by a triple combination with a potassium-sparing diuretic, especially since the activation of thiazide-sensitive NCC during LD treatment is partially aldosterone-induced [42,43].

### 3.4. Thiazide Diuretics

Thiazide diuretics are secreted into the *tubular* lumen *through* the organic anion secretory system and inhibit NCC, a Na/Cl cotransport on distal convoluted tubule, where up to 7% of the filtered sodium is reabsorbed. Nevertheless, blood pressure reduction persists even after volume depletion is balanced by compensatory mechanisms, which suggests a long-term effect on vascular resistances, probably by opening a calcium-activated potassium channel. Other mechanisms remain controversial: calcium antagonist activity, calcium desensibilization, or reverse whole-body regulation [46].

**Figure 3:**
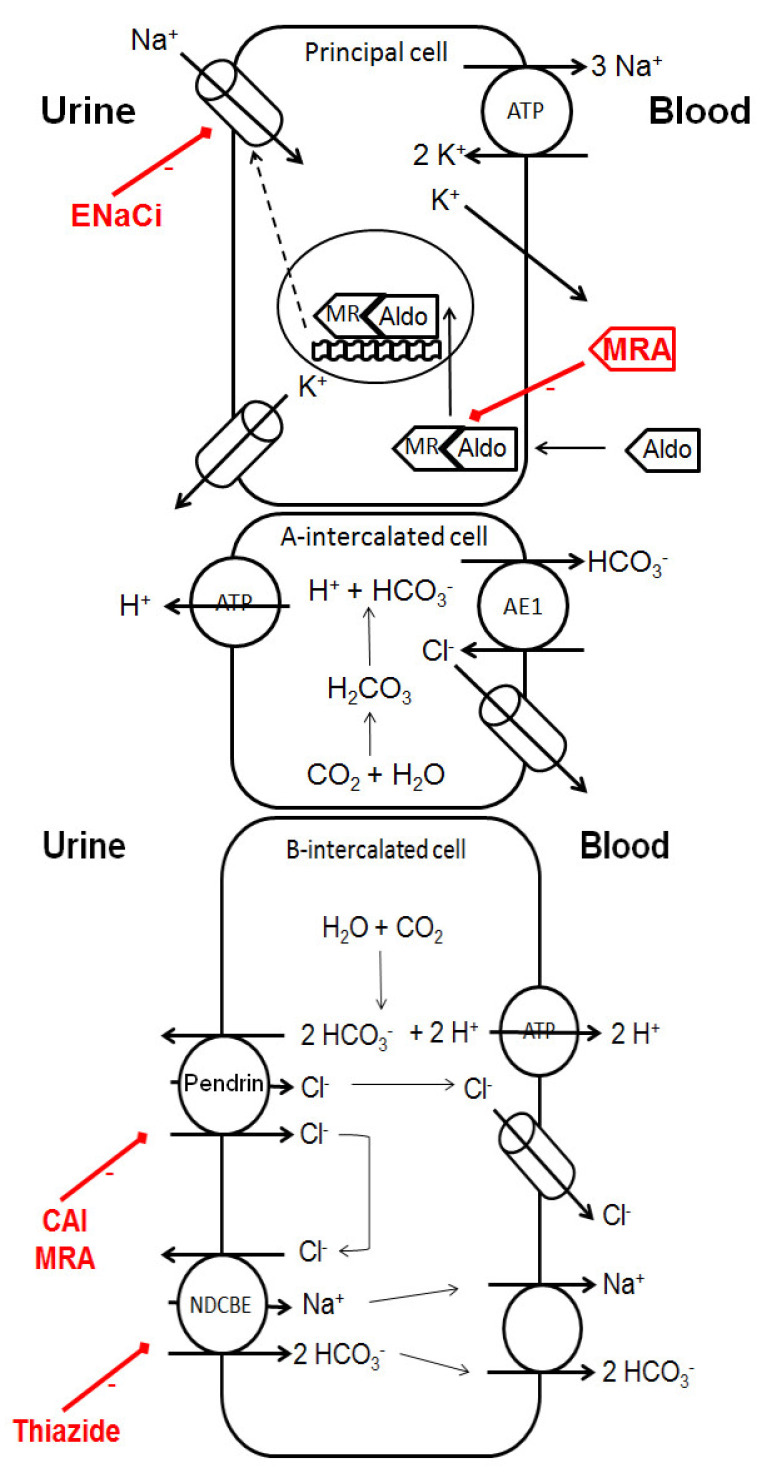
Effects of diuretics in the collecting duct. While mineralocorticoid receptor antagonists act through transcriptional mechanisms, ENaC inhibitors directly block sodium reabsorption. The potential effects of diuretics on B-intercalated cells are still only poorly described. Future studies may clarify their clinical implications. Aldo: aldosterone, MR: mineralocorticoid receptor, NDCBE: Na^+^-driven Cl^−^/HCO_3_^−^ exchanger, ENaC: epithelial sodium channel, MRA: mineralocorticoid receptor antagonists, ENaCi: epithelial sodium channel inhibitor, CAI: carbonic anhydrase inhibitor.

On the B-intercalated cells of the collecting duct, pendrin activation contributes to thiazide resistance [47], in addition to RAAS and sympathetic activation. ENaC expression is in turn fostered by pendrin activation. Thiazides may still have a significant natriuretic effect in advanced CKD, particularly in the case of LD resistance, and may inhibit Na+-driven chloride bicarbonate exchanger (NDCBE) in the collecting duct (Figure 3).

In sodium-sensitive hypertension, thiazides can decrease blood pressure enough to decrease intraglomerular pressure and thus the amount of filtered sodium, thereby allowing a new sodium balance [48]. However, in the case of RAAS and adrenergic activation, the thiazide-induced drop in blood pressure is insufficient to decrease the amount of filtered sodium. Thus, RAAS activation impairs blood pressure lowering in salt-resistant hypertension, so that association of thiazides with RAAS inhibitors is synergistic.

### 3.5. Mineralocorticoid Receptor Antagonists (MRA)

MRA are characterized by a slower onset of natriuresis, when compared to other diuretics. MRA act on basolateral side of aldosterone-sensitive cells of the collecting duct, and compete with aldosterone on its cytosolic receptor, thereby preventing its nuclear translocation. MRA thus lead to the proteasome-mediated degradation of ENaC (Figure 3). In principal cells, aldosterone stabilizes apical ENaC. Angiotensin II may restore aldosterone’s activity on the mineralocorticoid receptors of intercalated cells with the activation of pendrin-mediated sodium reabsorption in the case of volume depletion [49]. Other aldosterone effects include increased vascular tone and remodeling, increased response to catecholamine, and baroreflex modulation.

Spironolactone and eplerenone have a dose-dependent diuretic and potassium-sparing effect. These MRA showed reduced mortality in patients with heart failure with a reduced ejection fraction, irrespective of DM status [16,18]. Eplerenone also reduced major cardiovascular events in patients with acute myocardial infarction and left ventricular dysfunction [50]. The contribution of the natriuretic effect to this cardiovascular benefit is claimed to be marginal. However, as early as one month after starting MRA, body weight decreased more on eplerenone than on the placebo, and survival curves started to diverge, suggesting an early impact of MRA treatment [51], because of its diuretic effect and/or fewer hypokalemia-induced arrhythmias.

Finerenone, a non-steroidal MRA, reduced the risk of CKD progression and cardiovascular events in patients with T2D in addition to conventional RAAS blockade [19,20]. The benefit of finerenone in FIGARO-DKD was higher in the subgroup of patients receiving SGLT2i at baseline, with a 51% reduction of major adverse cardiovascular endpoints, compared to an 11% reduction in the absence of SGLT2i [20,52]. Indeed, MRA show a powerful synergy with all diuretics responsible for RAAS activation. Consequently, MRA are the cornerstone of aldosterone escape and resistant hypertension treatment [53].

Treatment with MRA on top of ACEI or ARB can be limited by hyperkalemia, more frequent in type 2 diabetes. Associations with LD, thiazides, or potassium binders may be required to prevent RAAS blockers discontinuation. SGLT2i may be useful to limit the rise of serum potassium, since SGLT2i have a kaliuretic effect by increasing flow in distal tubules [34]. Finerenone might induce less hyperkalemia and blood pressure decrease than spironolactone [54]. However, finerenone has no additional target to the mineralocorticoid receptor, and this excellent tolerance profile might be due to adequate dosing of MRA. Indeed, a modest natriuretic effect of SGLT2i is sufficient in patients with CKD to obtain a significant benefit for renal outcomes [55].

### 3.6. ENaC Inhibitors

ENaC inhibitors (amiloride, triamterene) directly block sodium channels in the principal cells of the collecting duct (Figure 3). In the case of heavy proteinuria, urokinase is responsible for the activation of abnormally filtered plasminogen. Resulting plasmin may ultimately increase ENaC activity [56]. ENaC inhibitors inhibit urokinase, oppose this key mechanism of aldosterone-independent proteinuria-induced sodium retention [57], and may prevent proteinuria-associated renal fibrosis. ENaC inhibitors could also inhibit proximal tubule Na/H exchangers.

The natriuretic effects of ENaC inhibitors and MRA are roughly equivalent: 2–4% of filtered sodium, or even more if sodium reabsorption is inhibited upstream (by loop or thiazide diuretics), or in the case of hypermineralocorticism-induced hypervolemia. ENaC inhibitors could be more efficient in edema resulting from nephrotic proteinuria with low RAAS activation. In those cases, sodium reabsorption is a consequence of the plasmin-induced increase in ENaC activity, rather than the aldosterone-induced increase in ENaC abundance. This mechanism might prove especially useful in patients with DKD and nephrotic syndrome. Conversely, ENaC inhibitors may be less effective with cirrhotic ascites with strong RAAS activation.

### 3.7. Natriuretic Peptides and Angiotensin Receptor-Neprilysin Inhibitor (ARNI)

Beneficial effects of natriuretic peptides [58] on the circulatory apparatus include: vasodilation, natriuresis, regulation of remodeling and energy homeostasis, RAAS [59] and sympathetic inhibition. BNP is produced in cardiac ventricles in response to fluid overload. However, BNP may fail to provide the expected protective effect in cardiorenal syndromes: RAAS and sympathetic activation may have a greater weight on the balance and part of the measured BNP may be less biologically active [58].

Sacubitril potentiates natriuretic peptides’ physiological increase by inhibiting neprilysin (NEP), the enzyme responsible for the inactivation of natriuretic peptides. In patients with DM, neprilysin seems to be upregulated, and response to natriuretic peptides is blunted [60]. Since NEP is also involved in angiotensin II inactivation and angiotensin 1–7 generation, RAAS blockers must be associated with ARNI. Otherwise, increased natriuretic peptides would be stultified by increased angiotensin II and decreased angiotensin 1–7 activities. The association of NEP inhibitors with ACEI increased the risk of angioedema because NEP is involved in bradykinin inactivation. A favorable benefit-risk balance has finally been obtained with sacubitril-valsartan.

Natriuretic peptides mainly increase natriuresis in proximal cortical tubule, but also in distal tubule with downregulation of ENaC, following an inhibited aldosterone and renin release. Natriuretic peptides also inhibit angiotensin II-induced release of vasopressin and angiotensin II effect on V2 receptors, increasing diuresis [58]. Natriuretic peptides increase GFR by vasodilating afferent arteriole and vasoconstricting efferent arteriole, and perhaps by directly increasing ultrafiltration coefficients, following the relaxation of mesangial cells. Therefore, natriuretic peptides lead to increased intraglomerular pressure, which could lead to increased proteinuria and renal failure progression that must be prevented by associated RAAS blockade. In fact, the sacubitril-induced increase in urinary albumin/creatinine ratio (UACR) is only partially inhibited by valsartan. In PARADIGM-HF, UACR was significantly higher at month 8 in the sacubitril-valsartan group compared to enalapril (mean difference: 0.3 mg/mmol, *p* < 0.001), [61] although the addition of a diuretic to RAAS inhibition should be associated with proteinuria decline [45]. This marginal UACR increase did not affect the slopes of eGFR decrease during the study: sacubitril-valsartan was associated with a slower rate of decline in GFR compared to enalapril, especially in patients with diabetes (−1.3 vs. −1.8 mL/min/1.73 m^2^/year; *p* < 0·0001) [60]. Overall, despite a marginal increase of albuminuria, sacubitril-valsartan may have net beneficial effects on heart and renal outcomes, by increasing natriuresis and vasodilating afferent arterioles without stimulating aldosterone synthesis [62].

Sacubitril-valsartan may contribute to improved glycemic control in patients with T2D, as it fosters the beneficial effect of natriuretic peptides on metabolism [63,64]. Moreover, sacubitril also inhibits the breakdown of many other peptides playing a beneficial role on glycemic status, including glucagon-like peptide 1 (GLP-1), bradykinin, and cyclic guanosine monophosphate (cGMP). The latter might modulate the tubuloglomerular feedback in the macula densa, and hinder sodium reabsorption in the proximal tubule through the sodium-hydrogen exchanger (NHE1). Additional studies in patients with renal failure and heavy proteinuria are needed to demonstrate its true nephroprotective effect.

### 3.8. Vasopressin Receptor Antagonist

In the collecting duct, vasopressin binds to its V2 receptors, thereby promoting the trafficking of aquaporins-2 (water channels) to the apical membrane. This mechanism allows water reabsorption following an osmotic gradient [65]. Tolvaptan is an antagonist of the V2 receptor. Accordingly, rather than a classical diuretic, tolvaptan more adequately qualifies as an aquaretic drug. Its benefit has been suggested for patients with polycystic kidney disease [66]. It may also prove useful in some cases of syndrome of inappropriate antidiuretic hormone secretion (SIADH) [67]. Unlike other diuretics, such as furosemide, tolvaptan does not seem to be affected by hypoalbuminemia [68].

The effect of tolvaptan on albuminuria is controversial and has been exclusively investigated in patients with polycystic kidney disease [69]. Treatment with tolvaptan may induce a slight, acute, and reversible decrease in GFR [70]. It is unclear whether this phenomenon arises from decreased intraglomerular pressure. Indeed, the albuminuric effect of vasopressin might result from increased glomerular leakage, mediated, inter alia, by RAAS, independently of changes in intraglomerular pressure [71].

Tolvaptan may help to alleviate fluid retention in patients with DM and nephrotic syndrome, but this benefit needs to be confirmed by further studies [72]. In fact, tolvaptan decreased neither the morbidity nor mortality of patients with HF in EVEREST (in which nearly half of the patients had DM), so that hyponatremia may not be a specific treatment target [73]. In the meantime, tolvaptan may be useful in some specific cases of resistant hyponatremia in patients with diabetes and severe fluid retention.

## 4. Aldosterone Breakthrough

Aldosterone levels may increase during RAAS blockade with ACEI or ARB in patients with DM. The incidence of aldosterone breakthrough is up to 53% after one year of RAAS blockade, with a liberate definition (any increase of serum aldosterone from baseline while on RAAS blockers) [74]. Its occurrence does not differ between ACEI or ARB, but is more likely when using short-acting RAAS blockers [57]. True aldosterone breakthrough may be defined as an increase of serum aldosterone greater than 10% over the baseline value. Using this stringent definition, we showed that aldosterone breakthrough still occurred in 28% of patients after one year in a large prospective cohort of ARB-treated patients with DM, [15] independently of RAAS blocker dosage. Intensive sodium depletion was a predictor of aldosterone breakthrough [15]. Therefore, increased dosage or association of diuretics without MRA in addition to RAAS blockade should foster aldosterone breakthrough. Further studies are required to explore the synergy between MRA and the other diuretics, including SGLT2i and ARNI.

Several negative outcomes could be expected after aldosterone breakthrough [15], including increased proteinuria and more rapid CKD progression [75]. However, we did not confirm these negative renal outcomes in the largest prospective study of patients with DM and overt nephropathy studied so far [15]. Dual RAAS blockade (losartan and lisinopril) better prevented renal failure progression than losartan alone in VA NEPHRON-D study at year 2, but this benefit vanished at study end [76]. We hypothesize that strong RAAS blockade led to aldosterone escape after year 2.

## 5. Practical Considerations

### 5.1. Preferential Indications of Each Diuretic

**Figure 4:**
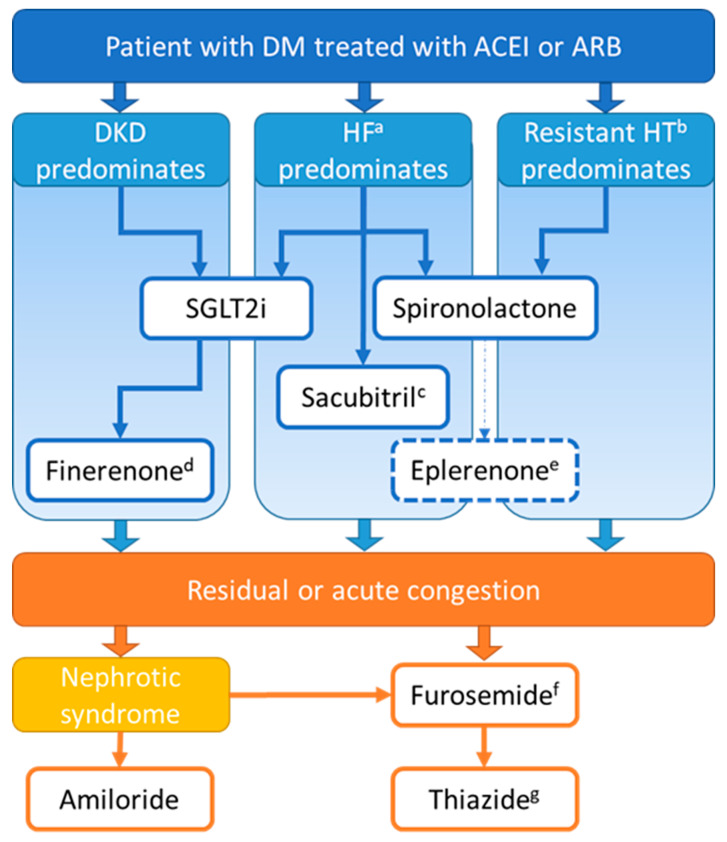
Suggested evidence-based approach for diuretic combinations in patients with diabetes mellitus treated with an angiotensin converting enzyme inhibitor or an angiotensin receptor blocker. a: especially with reduced ejection fraction. b: above-goal elevated blood pressure despite concurrent use of three antihypertensive drug classes (consider secondary hypertension assessment). c: discontinue ACEI or ARB before starting sacubitril/valsartan. d: if persistent proteinuria despite SGLT2i, consider adding finerenone. e: if endocrine adverse drug reactions are an issue, consider switching from spironolactone to eplerenone. f: for other loop diuretics; consider down-titrating to minimal effective dose after the emergency phase. g: e.g., hydrochlorothiazide or indapamide. DM: diabetes mellitus, ACEI: angiotensin converting enzyme inhibitor, ARB: angiotensin receptor blocker, DKD: diabetic kidney disease, HF: heart failure, HTA: hypertension.

The prescription of diuretics should be guided by the best available evidence, therefore following approved indications and existing guidelines. Moreover, the right diuretic combination should be wisely tailored according to each patient’s characteristics, comorbidities, and risk factors. It should be kept in mind that the benefit of most diuretics has been demonstrated in addition to RAAS blockers (ACEI or ARB), which are the cornerstone of nephroprotection in the setting of proteinuric kidney disease. Our suggested approach is depicted in Figure 4.

In most patients with T2D and established cardiovascular and/or kidney disease, SGLT2i are of utmost importance, and may be started on top of metformin regardless of glycemic control. This suggestion dovetails the recommendations of American and European learned societies [77]. On another note, sacubitril-valsartan occupies a prominent place in the treatment of chronic heart failure, irrespective of diabetes status [78].

Diuretics acting on the collecting tubule may be particularly useful in patients at risk of aldosterone breakthrough. In particular, finerenone might be a relevant addition to conventional RAAS blockade (with ACEI or ARB) and SGLT2i in patients with diabetic kidney disease [79]. Otherwise, spironolactone has proven its benefits in patients with HF with a reduced ejection fraction, and is also a relevant option when resistant hypertension is at the forefront. For its part, amiloride may prove useful when sodium retention is primarily driven by massive nephrotic-range proteinuria.

LD remain the handiest treatment in the case of residual or acute volume overload, due to their rapid onset of action and powerful natriuretic activity. If resistance to LD occurs, combination with thiazide diuretics can achieve efficient sequential nephron blockade. Nevertheless, because of the harmful counter-regulations arising from their use, LD should be promptly down-titrated to the minimal effective dose after the emergency phase.

### 5.2. Safe Prescription

Each diuretic comes with its own safety profile and array of adverse drug reactions (ADR), as summarized in the table. Combining low dose diuretics might alleviate the burden of each specific ADR compared to the prescription of full dose monotherapies, and improve drug acceptability. However, some ADR, shared by several diuretics, are subject to concerning additive or synergistic interactions.

Among those ADR, the risk of prerenal acute kidney injury following excessive sodium depletion is high, and common to all diuretics. Therefore, heightened caution is warranted when introducing and uptitrating diuretics, especially when combined. Extracellular volume status should be meticulously assessed before each diuretic escalation. In the case of normovolemia or hypovolemia, therapeutic escalation should be ideally postponed or associated with transient (or sustained) down-titration of LD. Likewise, patients should be educated to self-monitor blood pressure and weight, if necessary daily. Patients should also be taught to transiently discontinue their diuretic treatments in risky situations, such as diarrhea or significant decrease in dietary intakes. These simple measures may allow early warning in the case of excessive volume depletion, and may prevent the occurrence of prerenal failure.

Dyskalemia is another frequent concern in patients treated with diuretics. LD and thiazide diuretics may cause hypokalemia, while diuretics acting on the collecting duct increase the risk of hyperkalemia. Patients with DM are, per se, prone to hyperkalemia, especially when also treated with ACEI or ARB [80,81]. In this setting, the close monitoring of kalemia is required, all the more during uptitration of potassium-sparing diuretics. Moderate hyperkalemia (<5.5 mmol/L) might not always prompt diuretic discontinuation, given the benefit-risk ratio of RAAS blockade. Moderate hyperkalemia might rather encourage insistence on dietary rules and perhaps strengthen the medical treatment of hyperkalemia. Other options include combinations with diuretics known to decrease the risk of hyperkalemia, including SGLT2i.

## 6. Conclusions

We reviewed and discussed the available literature regarding the role of diuretics for cardioprotection and nephroprotection in patients with DM (including randomized clinical trials, reviews, and meta-analyses). Despite our best efforts, some studies may have escaped our scrutiny. Nonetheless, the antiproteinuric effect of each diuretic monotherapy encourages the benefit of their synergistic associations to be considered. Combined diuretics may help to overcome the escape to monotherapies. While adequately individualized associations may also decrease the risk of adverse drug reactions (such as dyskalemia), caution should be taken when introducing and titrating each different class to avoid massive natriuresis, excessive hypovolemia, and prerenal failure.

Achieving dry body weight is pivotal in patients with DM, since sodium retention occurs in diabetes at an early stage and a fortiori after the development of DKD and/or HF. Hypervolemia has detrimental effects on cardiovascular outcomes, and worsens DKD through uncontrolled proteinuria. Thiazides and LD were for a long time the main natriuretic drugs used to control this hypervolemia. However, sodium retention in patients with DM mainly occurs in proximal and distal convoluted tubules, where thiazides and LD increase sodium retention, and favor a deleterious aldosterone breakthrough. Therefore, diuretics acting on proximal tubules (ARNI and SGLT2i) and aldosterone blockade may be required in patients with DM. Additional studies are needed to assess whether proteinuria reduction translates to nephroprotection in diuretics other than finerenone or SGLT2i [7], while clarifying which synergistic strategies can best benefit patients with DM.

## Figures and Tables

**Table 1:** Comparison of different diuretic subclasses. Virtually all diuretics can precipitate prerenal acute kidney injury. PCT: proximal convoluted tubule, LH: loop of Henle, DCT: distal convoluted tubule, CD: collecting duct, Na: natremia, K: kalemia, Ca: calcemia, Mg: magnesemia, Urc: uricemia.

Subclass	Examples	Main Site of Action	Main Hydroelectrolytic Disorders	Main Other Adverse Drug Reactions
Carbonic anhydrase inhibitors	Acetazolamide	PCT	HypoK, hyperUrc	Urolithiasis
SGLT2 inhibitors	Dapagliflozin, canagliflozin, empagliflozin	PCT	/	Urogenital infections, euglycemic ketoacidosis
Loop diuretics	Furosemide, bumetanide	LH	HypoK, hypoCa, hypoMg, hyperUrc	Hypoacusis
Thiazide diuretics	Hydrochlorothiazide, indapamide	DCT	HypoNa, hypoK, hyperCa, hypoMg, hyperUrc	Skin tumors
Mineralocorticoid receptor antagonists	Spironolactone, eplerenone, finerenone	CD	HyperK	Gynecomastia
ENaC inhibitors	Amiloride	CD	HyperK	/
Neprilysin inhibitor	Sacubitril	PCT	(HyperK in association with valsartan)	Angioedema
Vasopressin receptor antagonist	Tolvaptan	CD	HyperNa, hyperUrc	Hepatotoxicity

## Data Availability

Not applicable.
